# Do we protect freshwater eels or do we drive them to extinction?

**DOI:** 10.1186/2193-1801-3-534

**Published:** 2014-09-16

**Authors:** Takaomi Arai

**Affiliations:** Institute of Oceanography and Environment, Universiti Malaysia Terengganu, Kuala Terengganu, 21030 Terengganu Malaysia

**Keywords:** *Anguilla*, Overfishing, Population, Stock assessment, Tropical eels

## Abstract

Freshwater eels are important animals because they have a unique catadromous life history and are used as food resources. European, American and Japanese eel populations now are considered to be outside the safe biological limits and are seriously threatened with extinction. Therefore, the European eel was recently categorised as critically endangered by the European Union and the United Nations. One of the reasons for the drastic decline in eel populations is overfishing, which has caused a high demand for eel aquaculture; eel aquaculture completely depends on wild juveniles, and in contrast to animals, artificial propagation has not yet succeeded for the eels. Therefore, commercial eel industries are now considering tropical eels as possible replacement for European and Japanese eels to compensate for declining stocks. In this study, I attempt to examine the present status of the biology and stock of tropical eels. However, useful scientific research and information on the biology and stock assessments of tropical eels are lacking, a situation quite different from that for other temperate freshwater eels, which have been well studied for several decades with trends and recruitment patterns being on record. Nevertheless, the present tropical eel catch has been reported as being less than half that of 20 years ago. The present trends in eel stocks and utilization for human consumption suggest that all eel populations will decline to numbers that fall outside safe biological limits and will be seriously threatened with extinction without protection and conservation from strict enforcement of local and international laws.

## Introduction

Freshwater eels are exotic animals, and despite a huge number of scientific studies conducted with eels, crucial aspects of their biology remain a mystery. No one has yet observed eels spawning in the natural environment, as spawning areas are located in the open ocean. This distinctly contrasts with other animals, such as anadromous salmon fish whose biology is well studied and better understood because localized spawning stocks are relatively easy to survey when the adults return to freshwater for spawning. Nineteen species of freshwater eels have been reported worldwide, 13 from tropical regions. Of the latter, seven species occur in the western Pacific around Indonesia (Ege 1939; Castle and Williamson 1974; Arai et al. 1999). Molecular phylogenetic researches on freshwater eels have recently revealed that tropical eels are the most basal species originating in the Indonesian region and that freshwater eels radiated out from the tropics to colonise the temperate regions (Minegishi et al. 2005). Tropical freshwater eels must be more closely related to the ancestral form than are their temperate counterparts.

Freshwater eels are the most important of the eel families from a conservation standpoint because they have a unique catadromous life history and are used as food resources. Recently, however, juvenile abundance has declined dramatically: by 99% for the European eel and by 80% for the Japanese eel (Dekker et al. 2003). Recruitment of the American eel near the species’ northern limit has virtually ceased (Dekker et al. 2003). Other eel species, including Australian and New Zealand eels (*Anguilla dieffenbachii* and *A. australis*), also show indications of decline (Dekker et al. 2003). The main problem is that all young eels used in cultivation are wild juveniles (glass eels and elvers), which are captured in estuaries. Almost all (90%) of the total world eel supply comes from aquaculture FAO (Food and Agriculture Organization of the United Nations) (2010). Therefore, the supply of eel resources for human consumption is completely dependent on wild catch.

The population size of wild European eel juveniles has linearly decreased from over 200 tonnes in the early 1960s to 20 tonnes at present, and in Japanese eels, a shortage of fry has become a serious problem for fish culture in recent years (Arai 2014a). Eel stocks throughout Europe are also declining (Dekker 2003a), and eel fishery yields have decreased in most European countries. The populations of the European, American and Japanese eels are considered to be outside safe biological limits, and current fisheries are not sustainable (Dekker 2003b; Dekker et al. 2003; Arai 2014a). The European eel was recently categorised as critically endangered by the European Union (EU) and the United Nations (CITES 2007), although other eels have not yet seriously been considered for protection. Since the early 1980s, juvenile recruitment has decreased, dropping to 1% of the levels encountered in the 1970s.

The causes of decline in stock and recruitment are not well understood. Overfishing, habitat loss and migration barriers, increased natural predation, parasitism, ocean climate variation, and pollution might have an impact (Knights 2003; Friedland et al. 2007; Bonhommeau et al. 2008; Marcogliese and Casselman 2009). Since the European eel was listed by CITES under Appendix II and came under protection in March 2009, and since the export/import ban was issued by the EU in 2010, the international trade of juvenile eels has changed. Most recently, the Japanese eel was added to the IUCN’s list with an endangered classification (IUCN 2014), suggesting it has a high risk of extinction. Species other than European and Japanese eels, including several tropical species, seem to have replaced the European eel on the international market. In addition, countries including Canada, the USA, the Dominican Republic, Morocco, Madagascar, the Philippines and Indonesia have now entered the market and supply juvenile eels for the farming industry in China, Japan, Taiwan and South Korea (Crook and Nakamura 2013; Anonymous 2013a, Anonymous 2014). However, fewer studies are available on tropical eels than for European, American, Japanese, Australian and New Zealand eels. The lack of availability of basic life history, stock and population information on the tropical eels could lead to further serious declines in these eels. Before tropical eel juveniles are used to replace and augment European and Japanese eels stocks, stock assessments and recruitment studies of source stocks are needed to determine the sustainability of tropical eels. However, consumers in East Asian countries do not pay attention to protection, conservation and enhancement of tropical eel populations, concentrating instead on having a stable eel supply and trade as they did with European and Japanese eels. If we continue such *ad hoc* eel resource usage, eels will become extinct around the world in the near future. Due to artificially induced breeding techniques for eel populations that are not yet firmly established, unlike populations of salmon, blue fin tuna and livestock, such a status will accelerate from threatened to declining wild eel stocks.

In this paper, I review the present status of recruitment and stocks of tropical eels. There is a clear lack of historical data regarding stock and recruitment of tropical eels. This lack of scientific research, assessment and protection will lead to the collapse of tropical eel populations and affect the maintenance of European, American, Japanese, Australian and New Zealand eels. Rapid stock assessment and continuous monitoring for recruitment in tropical eels are needed before usage of this resource to avoid eel extinctions around the world.

### Present status of trading in tropical freshwater eels

The present target tropical eel is *Anguilla bicolor* from Indonesia and the Philippines (Anonymous 2013a, Anonymous 2014). China, Japan, Taiwan and South Korea have imported cultured and sold the eel to consumers, using it to replace and compensate for declining European and Japanese eel sources. Although Indonesia and the Philippines prohibit the export of juvenile eels less than 150 g in weight from Indonesia and less than 15 cm in length from the Philippines to protect their resources, no regulations are enforced for juvenile fisheries in these countries (Anonymous 2012a, Anonymous 2012b). All marked eels are either wild-caught eels or cultured eels that were wild juveniles. No historical stock or juvenile recruitment data for eels are available in these countries; therefore, the fluctuation in the abundance of eels is not well understood.

### Present status of biological studies in tropical glass eels

**Figure 1:**
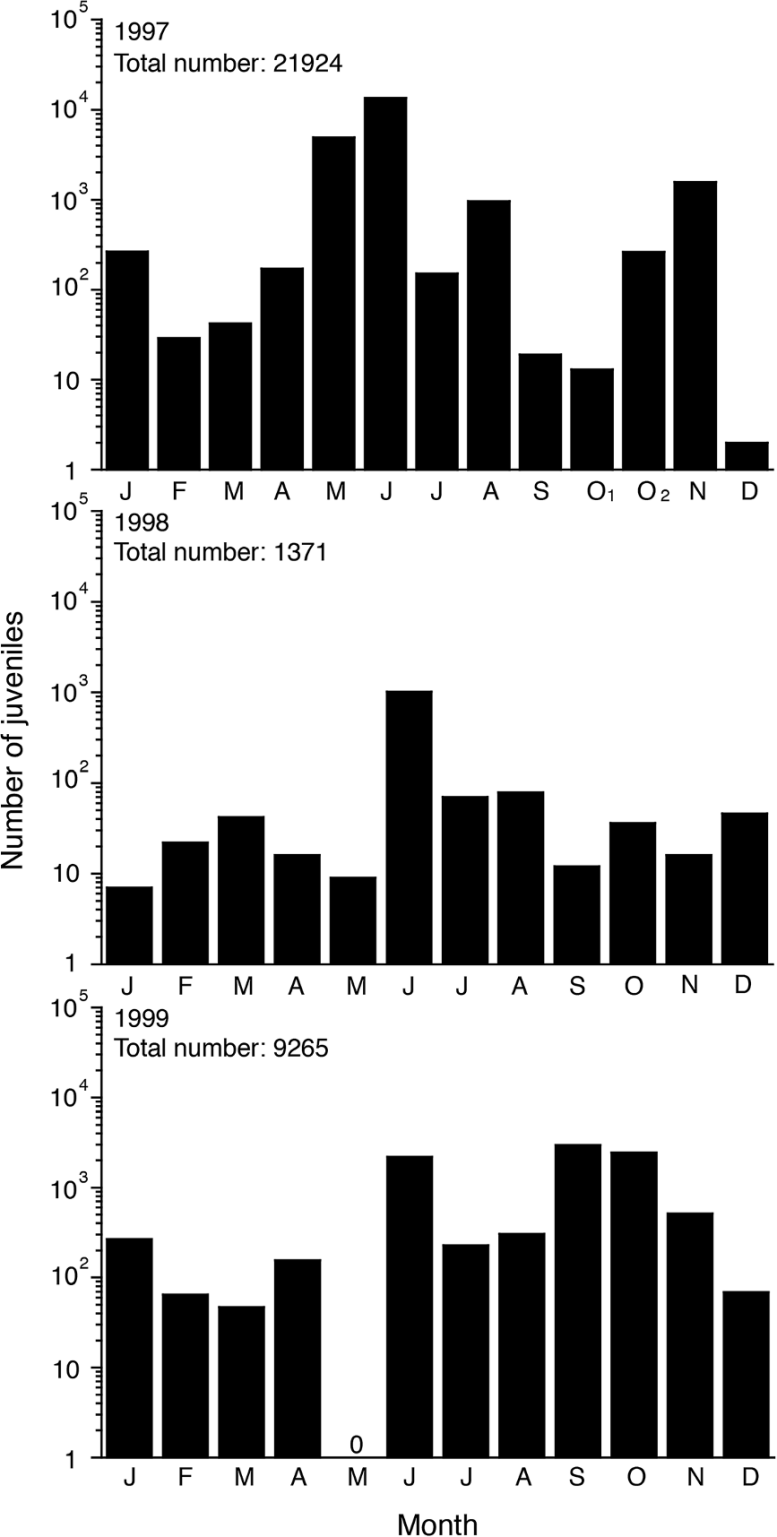
**Fluctuations of recruitment in tropical juvenile eels in Indonesia between 1997 and 1999.** Monthly abundance of 3 tropical juvenile eels collected at the new moon in the Poigar River estuary, north Sulawesi Island of Indonesia from 1997 to 1999 (for October 1997 samples: O_1_ = first new moon, O_2_ = second new moon) (Arai et al. 1999; Sugeha et al. 2001). Juvenile eels were collected at the mouth of the tropical river, and they were caught along a 10 m transect along the beach within 1.5 m from shore using 2 triangular scoop nets (mouth 0.3 m^2^, 1 mm mesh). The nets were fished simultaneously at depths of 25 to 50 cm in 10 replicate passes at hourly intervals (Arai et al. 1999; Sugeha et al. 2001). The temporal patterns of juvenile catches suggest tropical juveniles recruit to the estuary throughout the year with considerable inter-annual variation in the recruitment patterns. The recruitment patterns are clearly different from those of European, American, Japanese, Australian and New Zealand eels, which have much shorter seasonal ranges in recruitment period during about half the year or less (Matsui 1952; Haro and Krueger 1988; Gandolfi et al. 1984; Sloane 1984; Jellyman 1977). This figure was drawn using the original data from Arai et al. (1999) and Sugeha et al. (2001).

The only available data in tropical eels show the trend for recruitment for three years from 1997 to 1999 from quantitative sampling from an estuary in Indonesia (Arai et al. 1999; Sugeha et al. 2001). Juveniles were found to occur throughout each year (Figure 1), with the highest recruitment occurring at the time of the new moon (Sugeha et al. 2001). Although year-round recruitment in tropical freshwater eels was found in other regions such as Philippines (Tabeta et al. 1976), Australia (Shen and Tzeng 2007) and Taiwan (Leander et al. 2012), quantitative surveys adjusting moon phase or other ambient environmental parameters did not conduct in those studies. Thus, it seems to be impossible to understand the fluctuation of glass eel abundance exactly.

**Figure 2:**
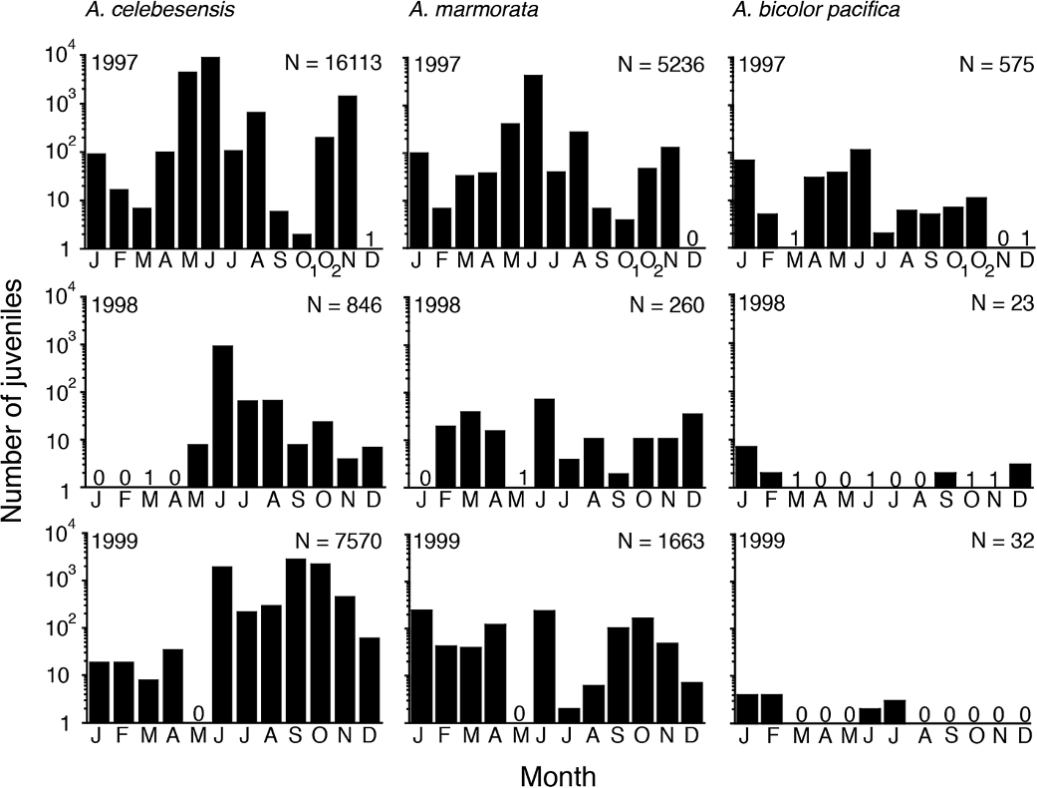
**Fluctuations of recruitment in tropical eels** ***Anguilla celebesensis*** **,** ***A. marmorata*** **and** ***A. bicolor pacifica*** **in Indonesia between 1997 and 1999.** Monthly abundance of glass eels of each species collected at new moon in the Poigar River estuary from 1997 to 1999 (for October 1997 samples: O_1_ = first new moon, O_2_ = second new moon). This figure was drawn using the original data from Arai et al. (1999) and Sugeha et al. (2001).

More than 30,000 glass eels were collected quantitatively in the Poigar River estuary on north Sulawesi Island, Indonesia, in monthly collections from 1997 to 1999 (Arai et al. 1999; Sugeha et al. 2001). The specimens were identified three species, *Anguilla celebesensis*, *A. marmorata*, and *A. bicolor pacifica*, were found each year in fluctuating abundances (Figure 2). *A. celebesensis* was the most abundant species and comprised 73.5%, 79.5%, and 81.9% of all glass eels recruiting to the estuary of the Poigar River in 1997, 1998, and 1999, respectively (Figure 2) (Arai et al. 1999; Sugeha et al. 2001). This species was relatively abundant in all three years with peaks during June in 1997 and 1998 and during September in 1999 (Figure 2). *A. marmorata* was the second most abundant species and comprised 23.8%, 18.8%, and 17.7% of the yearly catches, respectively, and reached peaks in abundance during June in 1997 and 1998, and during January in 1999 (Figure 2). *A. bicolor pacifica* comprised only 2.7%, 1.7%, and 0.3% of the yearly catches respectively, with peak catches in June in 1997, in January in 1998, and in January and February in 1999 (Figure 2). *A. celebesensis* and *A. marmorata* were collected almost throughout the year in 1997, 1998, and 1999, suggesting that in contrast to the temperate eels that recruit during half the year from winter to spring, these tropical eel species recruit to some degree throughout the year.

The temporal patterns of glass eels catches near the mouth of the Poigar River differed among species and years suggesting that there was considerable inter-annual variation in the recruitment patterns of glass eels in the region. However, such systematic surveys for tropical glass eels have never been conducted in other tropical regions. Further long-term surveys should be urgently needed to understand natural (e.g. ambient environments such as global climate change and oceanic transportation systems) and anthropogenic impacts (e.g. overexploitation, habitat degradation and pollution) on the recruitment of glass eels in tropical regions.

### Recruitment mechanisms of tropical freshwater eels

Glass eel catches in Japan has indicated that the catches decreased in association with El Niño events (Kimura et al. 2001). This suggests that the location of the spawning area changed with the appearance of El Niño. The salinity front functions are believed to be as a landmark for spawning migration of the Japanese eel (Kimura and Tsukamoto 2006). Indeed, many leptocephali have been distributed near the salinity front (Kimura et al. 1994, Tsukamoto et al. 2003). Associated with the El Niño events, salinity front moves considerably leading to change the leptocephali transportation route. The winter from December 1997 to February 1998 was recorded as great El Niño event, i.e. the period was the second warmest and seventh wettest since 1895 (Ross et al. 1998). If the spawning areas of tropical eels recruited to North Sulawesi move associated with movement of the salinity front around their spawning areas, transportation systems of tropical eel leptocephali might be also changed and the event lead to the low catch of glass eels in the estuary of the Poigar River. The spawning area of *A. marmorata* in the Poigar belonging to the North Pacific population (Minegishi et al. 2008) could be located in the western North Pacific where is the spawning ground of the Japanese eel *A. japonica* (Tsukamoto et al. 2011, Figure 1). Recently, one spawning-condition female *A. marmorata* was collected corresponded to the estimated spawning area in *A. marmorata* by Arai et al. (2002) as determined by otolith analyses. Thus, leptocephali of *A. marmorata* might be affected on the El Niño event in the winter between 1997 and 1998 in the western North Pacific. These results suggest the considerably low catch of glass eels in the Poigar River in 1998 in *A. marmorata* and similar declining of glass eels would be also found in *A. celebesensis* and *A. bicolor pacifica* in the year caused changes of transportation systems from their spawning grounds to growth habitats (Figures 1 and 2) due to passive transportation by oceanic currents from their spawning sites.

Although a few tropical glass eels recruited year round, temperate anguillid species showed much shorter seasonal ranges in recruitment period. In the northern hemisphere, *A. japonica* glass eels recruit to the east coast of Japan from October to May (Matsui 1952), the west coast of southern Japan from January to June (Kawakami et al. 1999) and the east coast of central Japan from November to June (Aoyama et al. 2012). For the Atlantic temperate eels, the peak in recruitment of *A. rostrata* has been reported to be in April and May (Haro and Krueger 1988), and the recruitment season of *A. anguilla* was from late November to early July, with the peak of upstream migration into freshwater in February and May (Gandolfi et al. 1984). In the southern hemisphere, on several streams in eastern Tasmania, the glass eels of *A. australis* were collected at the first riffle during all seasons of the year except mid summer with peak in October, and during late summer and autumn for the more tropical *A. reinhardtii* glass eels with peak in April (Sloane 1984). Jellyman (1977) found that the invasion by both New Zealand species into the Makara Stream commenced in July, with that of the long-finned eel *A. dieffenbachii* finishing by November with peaks in August and September, and that of the short-finned eel *A. australis schmidtii* finishing by December with peaks in September and October. This was similar to the seasonal migration of both species in the Waikato River, New Zealand, where the main migratory period extended from August to October (Jellyman 1979). These results suggest that tropical glass eels tend to recruit throughout the year while most temperate species recruit during about half of the year or less.

Analyses of the otolith microstructure showed that the ages at recruitment of tropical eels were constant throughout the year (Arai et al. 2001). The spawning seasons of tropical eels were found to extend throughout the year (Arai et al. 2001). The year-round spawning of tropical species and constant larval growth throughout the year extend the period of recruitment to estuarine habitats to year-round in tropical eels. Local short-distant migration made by tropical eels (Arai 2014b, Figure 3) enables such spawning ecology and recruitment mechanisms. For temperate eels, the retention of their spawning areas in the tropics would require that the eels migrate thousands of kilometres to have clearly seasonal patterns of downstream migration, spawning in the open ocean, and recruitment of glass eels.

**Figure 3:**
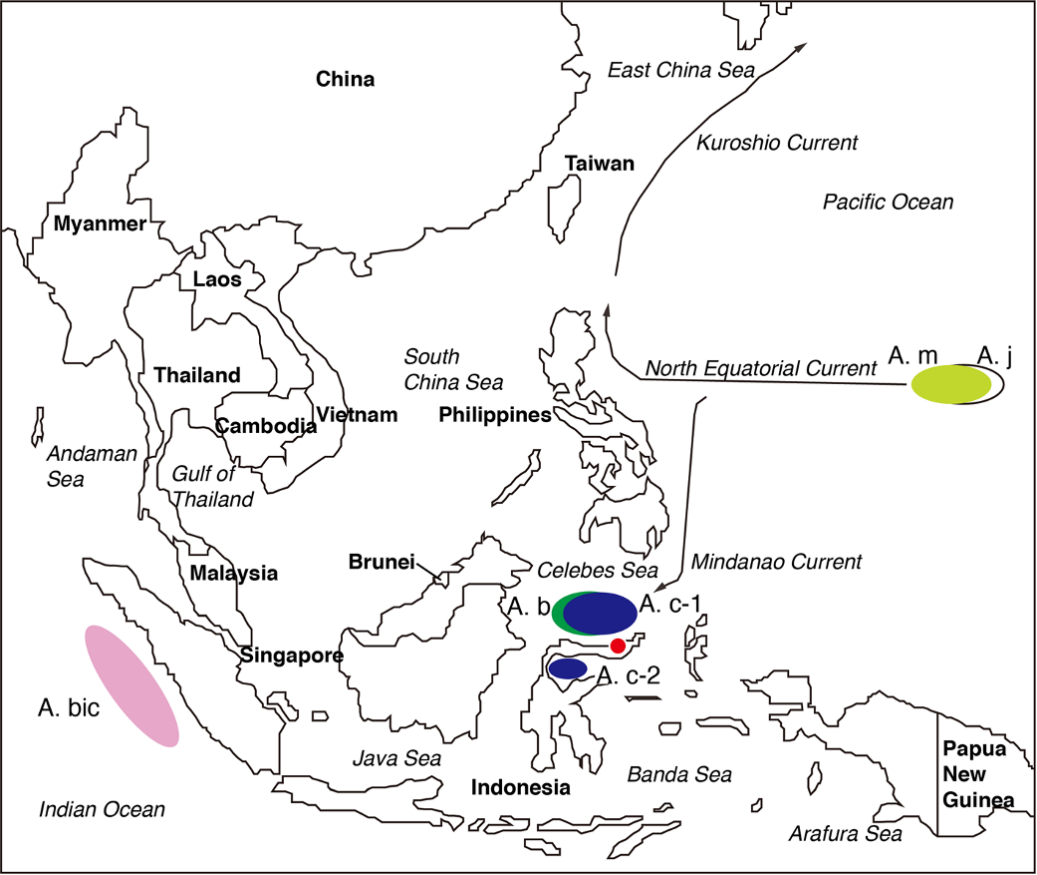
**Map of discovered spawning areas of tropical anguillid eels.** A spawning area of *Anguilla bicolor bicolor* locates near the Mentawai Trench off Sumatra Island of Indonesia (A. bic). The spawning areas of *A. celebesensis* (A. c-1) and *A. borneensis* (A. b) found in the Celebes Sea off North Sulawesi Island of Indonesia (Jespesen 1942; Aoyama et al. 2003). Another spawning area of *A. celebesensis* was further found in Tomini Bay off Central Sulawesi Island of Indonesia, central Sulawesi of Indonesia (A. c-2, Aoyama et al. 2003, Arai 2014b). The location of the spawning area of *A. marmorata* is in the western North Pacific (A. m, Arai et al. 2002; Tsukamoto et al. 2011) where is the spawning ground of the Japanese eel *A. japonica* (A. j, Tsukamoto et al. 2011). The oceanic currents from the spawning ground to growth habitats in *A. marmorata* are illustrated. A red spot indicates the study site of the tropical glass eel recruitments in the Poigar River estuary, north Sulawesi Island of Indonesia from 1997 to 1999 (Arai et al. 1999; Sugeha et al. (2001).

### Present status of stocks in temperate freshwater eels

**Figure 4:**
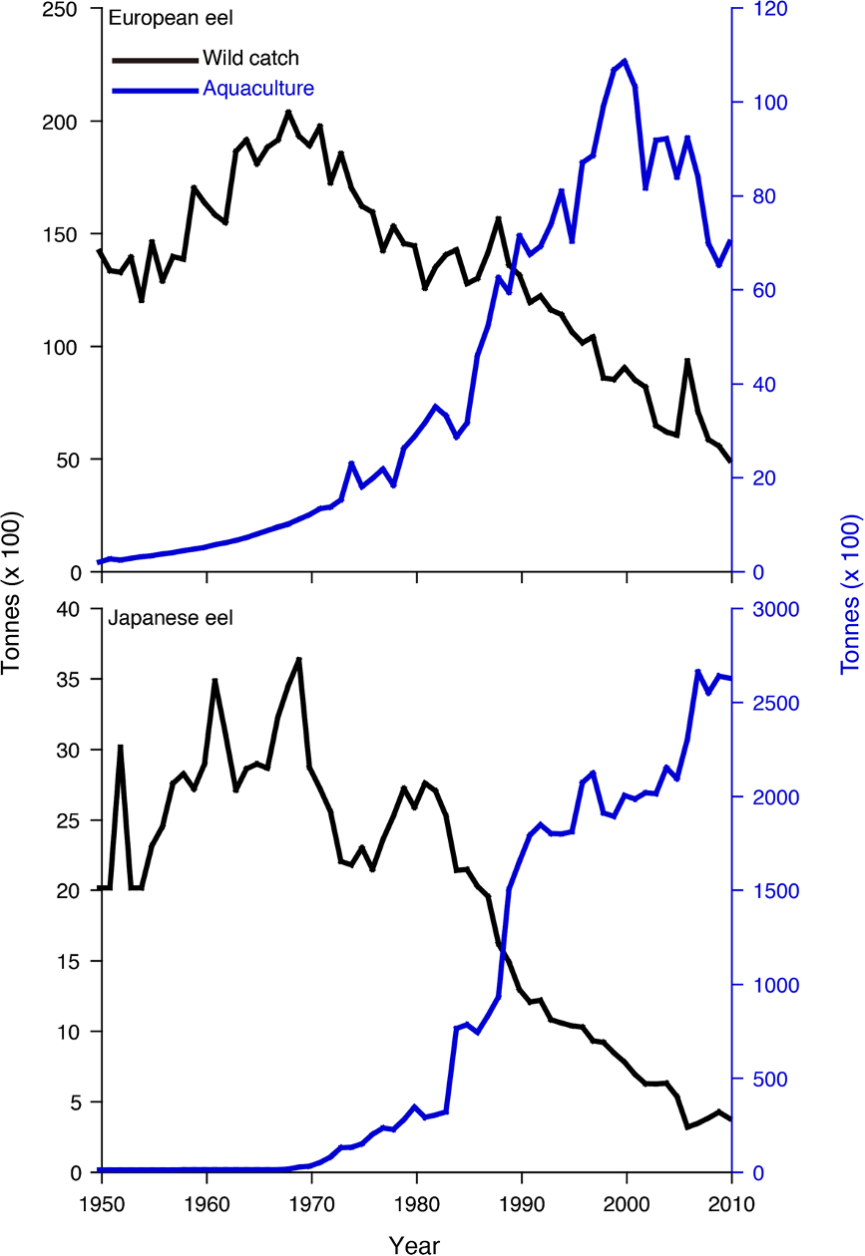
**Trends in global capture and aquaculture production between 1950 and 2010 for the European eel (top) and Japanese eel (bottom).** Sharp declines in wild European and Japanese eel populations correspond to drastically increased aquaculture demands for these eels after the 1970s. The peak capture of the Japanese eels is less than the lowest captures of European eels, indicating a relatively low virgin biomass of the Japanese eels. However, demand for aquaculture of *Anguilla japonica* is much more than that of *A. anguilla*, also indicating a vulnerability of the Japanese eel stock. This figure was drawn using the FAO FishFinder by the Food and Agriculture Organization of the United Nations (http://www.fao.org/fishery/fishfinder/contacts/en).

**Figure 5:**
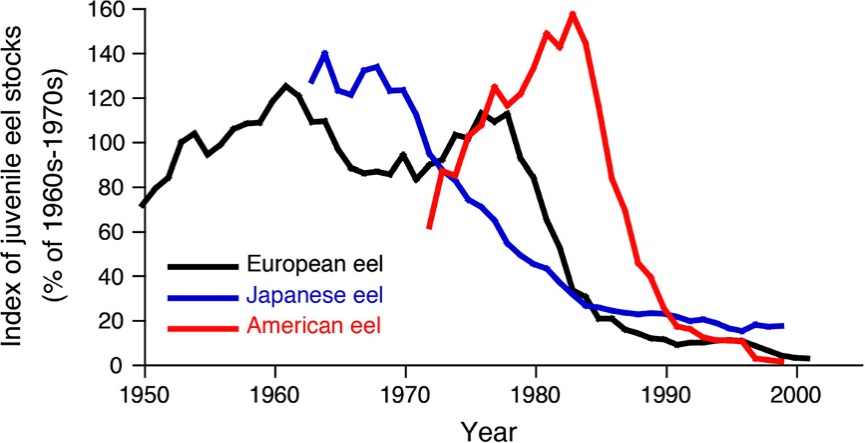
**Trends in juvenile stocks of the European, American and Japanese eels.** (Dekker et al. 2003). The data for European and Japanese eels are shown as landings of juveniles in each area, and for American eel is recruitment data to Lake Ontario at the northern limit of its distribution. Abundances of all juvenile eels are sharply declining after peaks; the European eel has declined by 99%, the Japanese eel by 80%, and recruitment of the American eel has virtually ceased. This figure references material from Dekker et al. (2003) and was drawn using original data provided by Dr. Willem Dekker.

In contrast to the tropical eels, historical stock data for wild eels are available for European, American, Japanese, Australian and New Zealand eels. For European and Japanese eels, wild catches fell gradually after the peak levels of the late 1970s and early 1980s in accordance with the increasing demand for eels in aquaculture (Figure 4). Trends in juvenile abundance of the major eel stocks for European, American and Japanese eels also suggest that juvenile populations have declined dramatically and clearly lie outside of safe biological limits (Figure 5). Recruitment of European and Japanese eels in each distribution range declined by 99% and 80%, respectively. Recruitment of American eel at the northern limit of its distribution has ceased (Figure 5).

### Worldwide decline of freshwater eel populations

The worldwide decline of freshwater eel populations is a major concern for animal conservation and diversity. European, American and Japanese eels have experienced sharp declines across their ranges over the last 30–40 years (ICES 2006; Aprahamian et al. 2007; Castonguay et al. 1994; Dekker et al. 2003, 2007) (Figures 4 and 5). In spite of the seriousness of the situation for juvenile eel recruitment, eel consumption is still increasing. To continue to supply large amounts of eels to consumers, the replacement and compensation have started to import eels from foreign countries, mainly the Philippines, Indonesia and Madagascar (Anonymous 2013a, [b], 2014). The main problem with consumption of this animal is that artificial propagation has not yet succeeded as it has with other common animals, such as salmon, blue fin tuna and livestock; therefore, juvenile eels are high-value aquaculture commodities that put high fishing pressure on a natural environment. Almost all (90%) of the world’s eel supply comes from aquaculture FAO (Food and Agriculture Organization of the United Nations) (2010) and the present eel aquaculture completely (100%) depends on wild juveniles. More than 90% of the world production of eels is cultured in East Asia, primarily Japan, Taiwan and China (Ringuet et al. 2002). Thus, wild juvenile eel catch will be needed in the future for these countries due to the increasing demands of aquaculture (Figure 4). To enhance natural eel stocks and continue their commercial usage for human consumption, studies related to the establishment of commercial juvenile production are urgently required and should focus on this goal as a means of protecting wild eel stocks.

### Lack of stock assessment and stock enhancement in freshwater eels

For the European eel, as a consequence of these concerns, the European Commission has agreed to an eel recovery plan, the aim of which is to return the European eel stock to sustainable levels of adult abundance and juvenile recruitment (Svedäng and Gipperth 2012). In 2007, the European eel was listed in Appendix II of CITES (the Convention on International Trade in Endangered Species of Wild Fauna and Flora) and Appendix II “includes species not necessarily threatened with extinction, but in which trade must be controlled to avoid utilization incompatible with their survival” (CITES 2007). Although stock assessment and management of the European eel have received increasing attention from both the scientific community and fisheries agencies in recent years (ICES 2006), such assessment and management of the Japanese eel have not yet been well studied. Such studies would help with the development of a concrete conservation policy and management applications for stock enhancement. Please note that despite the high demand for the product, the peak capture of Japanese eels is less than the lowest captures of European eels (Halpin 2007, Arai 2014a) (Figure 4). This fact indicates a relatively low virgin biomass of Japanese eels. To make matters worse, trade in tropical eels started with no scientific assessment and management before usage in spite of our experience with severely declining stocks in European, American, Japanese, Australian and New Zealand eels. Until now, there has been no information available on historical fishing records in tropical eels and only limited biological information compared with European, American, Japanese, Australian and New Zealand eels.

For marine species, dispersal during the larval phase is often important in shaping population genetic structure, and ocean currents play an important role in larval transport (Sponaugle et al. 2002; Fisher 2005). The migration distances for temperate eels such as the Atlantic and Japanese eels show thousands of km, while those of the tropical eels have been found local-short distance migration less than 100 km (Arai 2014b). The larval dispersal times for temperate eels such as the Atlantic, Australian, Japanese and New Zealand eels were 1 to 5 months longer than those of the tropical species (Arai et al. 2001). The time and distance required for dispersing larvae and migrating adults might be longer in temperate eels. The results suggest that higher genetic differentiation might be occurred in temperate species than that of tropical species.

Understanding the population structure of freshwater eels is the first step towards establishing management and conservation efforts for them. Most studies on the population genetic structure of anguillid eels have focused on the temperate species of *A. japonica* (Sang et al. 1994; Tseng et al. 2006; Han et al. 2008), *A. rostrata* (Avise et al. 1986; Wirth and Bernatchez 2003), *A. anguilla* (Lintas et al. 1998; Wirth and Bernatchez 2003; Dannewitz et al. 2005) and *A. australis* (Dijkstra and Jellyman 1999; Shen and Tzeng 2007). Several studies have examined the tropical species of *A. marmorata*, *A. bicolor* and *A. bengalensis* which have been found to have multiple populations or metapopulations throughout its wide range (Minegishi et al. 2008; Gagnaire et al. 2011; Donovan et al. 2012, Watanabe et al. 2014).

The Japanese eel shows spatial genetic differentiation throughout its range (Tseng et al. 2006), however Han et al. (2008) found that genetic differentiation is temporally stable at a single location. No apparent loss of genetic diversity and occurrence of a genetic bottleneck for the Japanese eel populations were found with estimating the effective population size (*Ne*) exceeded 500 (Han et al. 2008). In the European eel, highly significant genetic differentiation among inter-annual or among intra-annual arrival recruits was found (Pujolar et al. 2006), indicating variance in reproductive success (genetic patchiness). Because tropical eel species might have lower genetic differentiation made by local-short distance migration, loss of genetic diversity and occurrence of a genetic bottleneck would be occurred more frequently than those of temperate species. However, such evaluations have not been well examined in tropical species. Further molecular genetic study is needed for tropical eels and it would contribute to their sustainable management and conservation.

### Do we drive freshwater eels to extinction?

Although European, American, Japanese, Australian and New Zealand eels appeared to have much shorter seasonal ranges during the recruitment period for about half of the year or less (Matsui 1952; Haro and Krueger 1988; Gandolfi et al. 1984; Sloane 1984; Jellyman 1977), at least a few juveniles of the tropical eels recruited year round. The temporal pattern of tropical juvenile recruitment was found to have considerable interannual variation (Arai et al. 1999; Sugeha et al. 2001) (Figures 1 and 2). Thus, continuous long-term research is needed to determine the causes of the variation. Such year-round recruitment in tropical eels might be more convenient in aquaculture, which would be able to culture eels throughout year. In fact, 70 tonnes of eels were exported to Japan from one eel farm in Indonesia in 2013, and this amount is estimated to double in 2014 (Anonymous 2014). Because the present market price of juvenile eels is 150 times higher than 20 years ago, a number of village people near juvenile eel fishing grounds in Indonesia tend to concentrate on eel fishing only, whereas they used to focus on farming and fishing (Anonymous 2014). However, the juvenile eel catch is now reported to be half that of 20 years ago (Anonymous 2014), although the estimated decline has never been evaluated based on scientific research. The causes of decline in eel stocks and recruitment are not well understood. One of the main reasons must be overfishing, as sharp declines in wild European and Japanese eel populations correspond to drastically increased aquaculture demands for these eels since the 1970s (Figure 4). Now, tropical eels may have begun to follow the same trends as the European and Japanese eels. This suggests that we cannot rule out overfishing in tropical countries. Thus, if the wild juvenile eel catch of tropical eels continues to increase without assessment and protection of the stock and regulation of the catchment, all eel populations will decline to numbers outside safe biological limits. Currently, European, American and Japanese eels are seriously threatened with extinction due to eel consumption (Figures 4 and 5), and demand is still increasing. After the stocks and recruitment collapse in the present target eel species and areas, we will have to seek other targets for replacement and compensation to continue eel consumption. We may not be able to see such a unique animal on the earth in the near future.

## References

[CR1] (2012). Larangan pengeluaran benih sidat (Anguilla spp) dari wilayah negara Republik Indonesia keluar wilayah Negara Republik Indonesia.

[CR2] (2012). Reinstating the ban on the export of elvers. Department of Agriculture, Republic of the Philippines, Fisheries Administrative Order No. 242,.

[CR3] (2013). Competition on eel fishery and trading in Asia.

[CR4] (2013). The danger zone for eel.

[CR5] (2014). Eel battle in tropical area.

[CR6] Aoyama J, Shinoda A, Yoshinaga T, Tsukamoto K (2012). Late arrival of *Anguilla japonica* glass eels at the Sagami River estuary in two recent consecutive year classes: ecology and socio-economic impacts. Fish Sci.

[CR7] Aprahamian MW, Walker AM, Williams B, Bark A, Knights B (2007). On the application of models of European eel (*Anguilla anguilla*) production and escapement to the development of Eel Management Plans: the River Severn. ICES J Mar Sci.

[CR8] Arai T (2014). How have spawning ground investigations of the Japanese eel *Anguilla japonica* contributed to the stock enhancement?. Rev Fish Biol Fisheries.

[CR9] Arai T (2014). Evidence of local short-distance spawning migration of tropical freshwater eels, and implications for the evolution of freshwater eel migration. Ecol Evol.

[CR10] Arai T, Aoyama J, Limbong D, Tsukamoto K (1999). Species composition and inshore migration of the tropical eels *Anguilla* spp. recruiting to the estuary of the Poigar River, Sulawesi Island. Mar Ecol Prog Ser.

[CR11] Arai T, Limbong D, Otake T, Tsukamoto K (2001). Recruitment mechanisms of tropical eels, *Anguilla* spp., and implications for the evolution of oceanic migration in the genus *Anguilla*. Mar Ecol Prog Ser.

[CR12] Arai T, Marui M, Miller MJ, Tsukamoto K (2002). Growth history and inshore migration of the tropical eel, *Anguilla marmorata* in the Pacific. Mar Biol.

[CR13] Avise JC, Helfman GS, Sauders NC, Hales LS (1986). Mitochondrial DNA differentiation in North Atlantic eels: population genetic consequences of an unusual life history pattern. Proc Natl Acad Sci U S A.

[CR14] Bonhommeau S, Chassot E, Rivot E (2008). Fluctuations in European eel (*Anguilla anguilla*) recruitment resulting from environmental changes in the Sargasso Sea. Fish Oceanogr.

[CR15] Castle PHJ, Williamson GR (1974). On the validity of the freshwater eel species *Anguilla ancestralis* Ege from Celebes. Copeia.

[CR16] Castonguay M, Hodson PV, Moriarty C, Drinkwater KF, Jessop BM (1994). Is there a role of ocean environment in American and European eel decline. Fish Oceanogr.

[CR17] (2007). Proposal: Inclusion of *Anguilla anguilla* (L). in Appendix II in accordance with Article II §2(a). Convention on International Trade in Endangered Species of wild flora and fauna.

[CR18] Crook V, Nakamura M (2013). Glass eels: Assessing supply chain and market impacts of a CITES listing on *Anguilla* species. Traffic Bull.

[CR19] Dannewitz J, Maes GE, Johansson L, Wickström H, Volckaert FAM, Järvi T (2005). Panmixia in the European eel: a matter of time. Proc Roy Soc B.

[CR20] Dekker W (2003). On the distribution of the European eel (*Anguilla anguilla*) and its fisheries. Can J Fish Aquat Sci.

[CR21] Dekker W (2003). Did lack of spawners cause the collapse of the European eel, *Anguilla anguilla*?. Fish Manag Ecol.

[CR22] Dekker W, Pawson M, Wickström H (2007). Is there more to eels than slime? An introduction to the papers presented at the ICES Theme Session in September 2006. ICES J Mar Sci.

[CR23] Dekker W, Casselman JM, Cairns DK, Tsukamoto K, Jellyman D, Lickers H, many others (2003). Québec Declaration of Concern: worldwide decline of eel resources necessitates immediate action. Fisheries.

[CR24] Dijkstra LH, Jellyman DJ (1999). Is the subspecies classification of the freshwater eels *Anguilla australis australis* Richardson and *A. a. schmidtii* Phillipps still valid?. Mar FreshwRes.

[CR25] Donovan S, Pezold F, Chen Y, Lynch B (2012). Phylogeography of *Anguilla marmorata* (Teleostei: Anguilliformes) from the eastern Caroline Islands. Ichthyol Res.

[CR26] Ege VA (1939). Revision of the Genus *Anguilla* Shaw. Dana Rep.

[CR27] (2010). The state of world fisheries and aquaculture.

[CR28] Fisher R (2005). Swimming speeds of larval coral reef fishes: impacts on self-recruitment and dispersal. Mar Ecol Prog Ser.

[CR29] Friedland KD, Miller MJ, Knight B (2007). Oceanic changes in the Sargasso Sea and declines in recruitment of the European eel. ICES J Mar Sci.

[CR30] Gagnaire PA, Minegishi Y, Zenboudji S, Valade P, Aoyama J, Berrebi P (2011). Within-population structure highlighted by differential introgression across semipermeable barriers to gene flow in *Anguilla marmorata*. Evolution.

[CR31] Gandolfi G, Pesaro M, Tongiorgi P (1984). Environmental factors affecting the ascent of elver, *Anguilla anguilla* (L) into the Arno River. Oebalia.

[CR32] Halpin P (2007). Unagi: Freshwater “Eel”. Anguilla japonica, A. anguilla, A. rostrata.

[CR33] Han YS, Sun YL, Liao YF, Shen KN, Liao IC, Tzeng WN (2008). Temporal analysis of population genetic composition in the overexploited Japanese eel *Anguilla japonica*. Mar Biol.

[CR34] Haro AJ, Krueger WH (1988). Pigmentation, size, and migration of elvers *Anguilla rostrata* (Lesueur) in a coastal Rhode Island stream. Can J Zool.

[CR35] (2006). Report of the Joint EIFAC/ICES Working Group on Eels. Rome, 23-27 January 2006. EIFAC Occasional Paper. No. 38, ICES CM 2006/ACFM:16. Rome, FAO/Copenhagen, ICES. 2006. 352pp.

[CR36] (2014). The IUCN Red List of Threatened Species. Version 2014.2.

[CR37] Jellyman DJ (1977). Invasion of a New Zealand freshwater stream by glass eels of two *Anguilla* spp. New Zealand J Mar Freshwat Res.

[CR38] Jellyman DJ (1979). Upstream migration of glass-eels (*Anguilla* spp.) in the Waikato River. NZ J Mar Freshw Res.

[CR39] Kawakami Y, Mochioka N, Nakazono A (1999). Immigration patterns of glass-eels *Anguilla japonica* entering river in northern Kyushu Japan. Bull Mar Sci.

[CR40] Kimura S, Inoue T, Sugimoto T (2001). Fluctuation in the distribution of low salinity water in the North Equatorial Current and its effect on the larval transport of the Japanese eel. Fish Oceanogr.

[CR41] Kimura S, Tsukamoto K (2006). The salinity front in the North Equatorial Current: a landmark for the spawning migration of the Japanese eel (*Anguilla japonica*) related to the stock recruitment. Deep-Sea Res II.

[CR42] Kimura S, Tsukamoto K, Sugimoto T (1994). A model for the larval migration of the Japanese eel: roles of the trade winds and salinity front. Mar Biol.

[CR43] Knights B (2003). A review of the possible impacts of long-term oceanic and climate changes and fishing mortality on recruitment of anguillid eels of the Northern Hemisphere. Sci Total Environ.

[CR44] Leander NJ, Shen KN, Chen RT, Tzeng WN (2012). Species composition and seasonal occurrence of recruiting glass eels (*Anguilla* spp.) in the Hsiukuluan River, Eastern Taiwan. Zool Stud.

[CR45] Lintas C, Hirano J, Archer S (1998). Genetic variation of the European eel (*Anguilla anguilla*). Mol Mar Biol Biotechnol.

[CR46] Marcogliese LA, Casselman JM (2009). Long-term trends in size and abundance of juvenile American eel (*Anguilla rostrata*) in the St. Lawrence River. Am Fishe Soc Symp.

[CR47] Matsui I (1952). Study on the morphology, ecology and pond culture of the Japanese eel (*Anguilla japonica* Temminck & Schlegel). J Shimonoseki Coll Fish.

[CR48] Minegishi Y, Aoyama J, Inoue JG, Miya M, Nishida M, Tsukamoto K (2005). Molecular phylogeny and evolution of the freshwater eels genus *Anguilla* based on the whole mitochondrial genome sequences. Mol Phylogen Evol.

[CR49] Minegishi Y, Aoyama J, Tsukamoto K (2008). Multiple population structure of the giant mottled eel *Anguilla marmorata*. Mol Ecol.

[CR50] Pujolar JM, Maes GE, Volckaert FAM (2006). Genetic patchiness among recruits in the European eel *Anguilla anguilla*. Mar Ecol Prog Ser.

[CR51] Ringuet S, Muto F, Raymakers C (2002). Eels, their harvest in Europe and Asia. Traffic Bull.

[CR52] Ross T, Lott N, McCown S, Quinn D (1998). National Climatic Data Center Technical Report No. 98–02.

[CR53] Sang TK, Chang HY, Chen CT, Hui CF (1994). Population structure of the Japanese eel, *Anguilla japonica*. Mol Biol Evol.

[CR54] Shen KN, Tzeng WN (2007). Genetic differentiation among populations of the shortfinned eel *Anguilla australis* from East Australia and New Zealand. J Fish Biol.

[CR55] Sloane RD (1984). Distribution, abundance, growth and food of freshwater eel (*Anguilla* spp) in the Dauglass River, Tasmania. Aust J Mar Freshwat Res.

[CR56] Sponaugle S, Cowen RK, Shanks A, Morgan SG, Leis JM, Pineda JS, Boehlert GW, Kingsford MJ, Lindeman KC, Grimes C, Munro JL (2002). Predicting self-recruitment in marine populations: biophysical correlates and mechanisms. Bull Mar Sci.

[CR57] Sugeha HY, Arai T, Miller MJ, Limbong D, Tsukamoto K (2001). Inshore migration of the tropical eels *Anguilla* spp. recruiting to the Poigar River estuary on north Sulawesi Island. Mar Ecol Prog Ser.

[CR58] Svedäng H, Gipperth L (2012). Will regionalization improve fisheries management in the EU? An analysis of the Swedish eel management plan reflects difficulties. Mar Pol.

[CR59] Tabeta O, Tanimoto T, Takai T, Matsui I, Imamura T (1976). Seasonal occurrence of anguillid elvers in Cagayan River, Luzon Island, the Philippines. Bull Japan Soc Sci Fish.

[CR60] Tseng MC, Tzeng WN, Lee SC (2006). Historical decline in the Japanese eel *Anguilla japonica* in northern Taiwan inferred from genetic variations. Zool Stud.

[CR61] Tsukamoto K, Otake T, Mochioka N, Lee TW, Fricke H, Inagaki T, Aoyama J, Ishikawa S, Kimura S, Miller MJ, Hasumoto H, Oya M, Suzuki Y (2003). Seamounts, new moon and eel spawning: the search for the spawning site of the Japanese eel. Environ Biol Fish.

[CR62] Tsukamoto K, Chow S, Otake T, Kurogi H, Mochioka N, Miller MJ, Aoyama J, Kimura S, Watanabe S, Yoshinaga T, Shinoda A, Kuroki M, Oya M, Watanabe T, Hata K, Ijiri S, Kazeto Y, Nomura K, Tanaka H (2011). Oceanic spawning ecology of freshwater eels in the western North Pacific. Nat Commun.

[CR63] Watanabe S, Miller MJ, Aoyama J, Tsukamoto K (2014). Evaluation of the population structure of *Anguilla bicolor* and *A. bengalensis* using total number of vertebrae and consideration of the subspecies concept for the genus *Anguilla*. Ecol Freshw Fish.

[CR64] Wirth T, Bernatchez L (2003). Decline of North Atlantic eels: a fatal synergy?. Proc Roy Soc B.

